# *N*′-(Furan-2-ylmethyl­ene)-2-hydroxy­benzohydrazide

**DOI:** 10.1107/S1600536808034636

**Published:** 2008-10-25

**Authors:** Yan-Xia Zhang

**Affiliations:** a Department of Materials Science and Chemical Engineering, Taishan University, 271021 Taian, Shandong, People’s Republic of China

## Abstract

In the title mol­ecule, C_12_H_10_N_2_O_3_, the aromatic and furan rings form a dihedral angle of 8.89 (1)° and an intra­molecular N—H⋯O hydrogen bond occurs. In the crystal structure, inter­molecular O—H⋯O hydrogen bonds link the mol­ecules into zigzag chains running along the *c* axis.

## Related literature

For background on Schiff bases, see: Garnovskii *et al.* (1993 ▶); Anderson *et al.* (1997 ▶); Musie *et al.*, (2001 ▶); Paul *et al.* (2002 ▶); Yang, (2006 ▶). For reference bond distances, see: Allen *et al.* (1987 ▶).
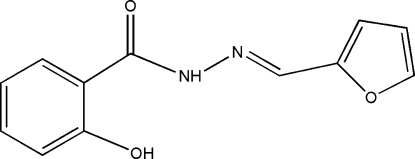


## Experimental

### 

#### Crystal data

C_12_H_10_N_2_O_3_
*M* *_r_* = 230.22
Monoclinic, 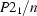

*a* = 4.9898 (5) Å
*b* = 20.662 (2) Å
*c* = 10.6994 (11) Å
β = 101.421 (2)°
*V* = 1081.24 (19) Å^3^
*Z* = 4
Mo *K*α radiation
μ = 0.10 mm^−1^
*T* = 295 (2) K
0.12 × 0.10 × 0.06 mm

#### Data collection

Bruker SMART APEXII area-detector diffractometer
Absorption correction: multi-scan (*SADABS*; Bruker, 2005 ▶) *T*_min_ = 0.98, *T*_max_ = 0.99
5631 measured reflections
1904 independent reflections
1451 reflections with *I* > 2σ(*I*)
*R*_int_ = 0.022

#### Refinement

*R*[*F* ^2^ > 2σ(*F* ^2^)] = 0.034
*wR*(*F* ^2^) = 0.097
*S* = 1.02
1904 reflections
156 parameters
H-atom parameters constrained
Δρ_max_ = 0.12 e Å^−3^
Δρ_min_ = −0.13 e Å^−3^

### 

Data collection: *APEX2* (Bruker, 2005 ▶); cell refinement: *SAINT* (Bruker, 2005 ▶); data reduction: *SAINT*; program(s) used to solve structure: *SHELXTL* (Sheldrick, 2008 ▶); program(s) used to refine structure: *SHELXTL*; molecular graphics: *SHELXTL*; software used to prepare material for publication: *SHELXTL*.

## Supplementary Material

Crystal structure: contains datablocks global, I. DOI: 10.1107/S1600536808034636/bg2220sup1.cif

Structure factors: contains datablocks I. DOI: 10.1107/S1600536808034636/bg2220Isup2.hkl

Additional supplementary materials:  crystallographic information; 3D view; checkCIF report

## Figures and Tables

**Table 1:** Hydrogen-bond geometry (Å, °)

*D*—H⋯*A*	*D*—H	H⋯*A*	*D*⋯*A*	*D*—H⋯*A*
O1—H1⋯O2^i^	0.82	2.14	2.804 (2)	139
N1—H1*A*⋯O1	0.86	1.99	2.650 (2)	133

Symmetry code: (i) 
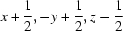
.

**Fig. 1.**
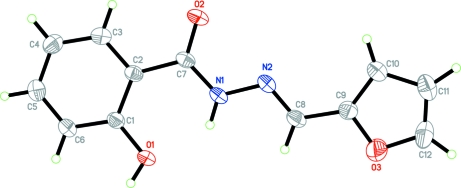
The molecular structure of (I), with displacement ellipsoids drawn at the 50% probability level.

**Fig. 2.**
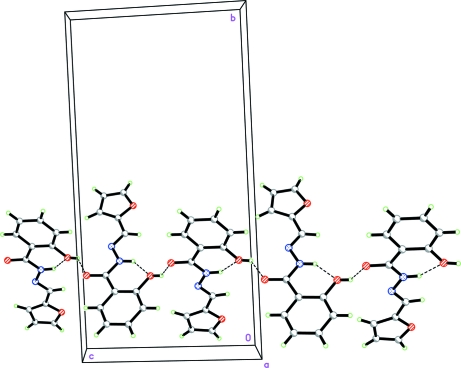
The structure of the infinite chains formed via hydrogen bonds, H atoms have been omitted for clarity. Dashed lines indicate hydrogen bonds.

## supplementary crystallographic information

### Comment

Recently, a number of Schiff-bases have been investigated because of their coordination chemistry (Garnovskii *et al.*, 1993; Musie *et al.*, 2001; Paul *et al.*, 2002; Yang, 2006;) and biological systems (Anderson *et al.*, 1997). In order to search for new Schiff-bases with higher bioactivity, the title compound, (I), was synthesized and its crystal structure determined. In (I) (Fig. 1), the bond lengths and angles are in good agreement with the expected values (Allen *et al.*, 1987). In the crystal structure (Fig. 2), the molecules are linked into infinite chains by O—H···O hydrogen bonds. There is also an intramolecular N—H···O hydrogen bond.

### Experimental

The title compound was synthesized by the reaction of 2-hydroxy-benzoic acid hydrazide(1 mmol, 152.2 mg) with furan-2-carbaldehyde(1 mmol, 96.2 mg) in ethanol(20 ml) under reflux conditions (348 K) for 5 h. The solvent was removed and the solid product recrystallized from tetrahydrofuran. After six days orange crystals suitable for the X-ray diffraction study were obtained.

### Refinement

All H atoms were placed in idealized positions (C—H = 0.93— 0.97 Å, N—H = 0.86 Å) and refined as riding atoms. For those bound to C, *U*_iso_(H) = 1.2 or 1.5*U*_eq_(C). while for those bound to N, *U*_iso_(H) = 1.2 *U*_eq_(N).

### Crystal data

**Table:**

C_12_H_10_N_2_O_3_	*F*(000) = 480
*M**_r_* = 230.22	*D*_x_ = 1.414 Mg m^−^^3^
Monoclinic, *P*2_1_/*n*	Mo *K*α radiation, λ = 0.71073 Å
Hall symbol: -P 2yn	Cell parameters from 1748 reflections
*a* = 4.9898 (5) Å	θ = 2.2–25.0°
*b* = 20.662 (2) Å	µ = 0.10 mm^−^^1^
*c* = 10.6994 (11) Å	*T* = 295 K
β = 101.421 (2)°	Block, orange
*V* = 1081.24 (19) Å^3^	0.12 × 0.10 × 0.06 mm
*Z* = 4	

### Data collection

**Table:**

Bruker SMART APEXII area-detector diffractometer	1904 independent reflections
Radiation source: fine-focus sealed tube	1451 reflections with *I* > 2σ(*I*)
graphite	*R*_int_ = 0.023
φ and ω scans	θ_max_ = 25.1°, θ_min_ = 2.0°
Absorption correction: multi-scan (SADABS; Bruker, 2005)	*h* = −5→5
*T*_min_ = 0.98, *T*_max_ = 0.99	*k* = −24→21
5631 measured reflections	*l* = −11→12

### Refinement

**Table:**

Refinement on *F*^2^	Secondary atom site location: difference Fourier map
Least-squares matrix: full	Hydrogen site location: inferred from neighbouring sites
*R*[*F*^2^ > 2σ(*F*^2^)] = 0.034	H-atom parameters constrained
*wR*(*F*^2^) = 0.097	*w* = 1/[σ^2^(*F*_o_^2^) + (0.0472*P*)^2^ + 0.1418*P*] where *P* = (*F*_o_^2^ + 2*F*_c_^2^)/3
*S* = 1.02	(Δ/σ)_max_ < 0.001
1904 reflections	Δρ_max_ = 0.12 e Å^−^^3^
156 parameters	Δρ_min_ = −0.12 e Å^−^^3^
0 restraints	Extinction correction: SHELXTL (Sheldrick, 2008), Fc^*^=kFc[1+0.001xFc^2^λ^3^/sin(2θ)]^-1/4^
Primary atom site location: structure-invariant direct methods	Extinction coefficient: 0.0107 (19)

### Special details

**Table:**

Geometry. All e.s.d.'s (except the e.s.d. in the dihedral angle between two l.s. planes) are estimated using the full covariance matrix. The cell e.s.d.'s are taken into account individually in the estimation of e.s.d.'s in distances, angles and torsion angles; correlations between e.s.d.'s in cell parameters are only used when they are defined by crystal symmetry. An approximate (isotropic) treatment of cell e.s.d.'s is used for estimating e.s.d.'s involving l.s. planes.
Refinement. Refinement of *F*^2^ against ALL reflections. The weighted *R*-factor *wR* and goodness of fit *S* are based on *F*^2^, conventional *R*-factors *R* are based on *F*, with *F* set to zero for negative *F*^2^. The threshold expression of *F*^2^ > σ(*F*^2^) is used only for calculating *R*-factors(gt) *etc*. and is not relevant to the choice of reflections for refinement. *R*-factors based on *F*^2^ are statistically about twice as large as those based on *F*, and *R*- factors based on ALL data will be even larger.

### Fractional atomic coordinates and isotropic or equivalent isotropic displacement parameters (Å^2^)

**Table:**

	*x*	*y*	*z*	*U*_iso_*/*U*_eq_	
O1	0.2921 (2)	0.22463 (6)	0.59528 (10)	0.0611 (4)	
H1	0.3615	0.2261	0.5320	0.092*	
O2	0.2021 (2)	0.22907 (5)	0.97330 (9)	0.0541 (3)	
O3	−0.6276 (3)	0.42331 (7)	0.68440 (12)	0.0815 (4)	
N1	0.0370 (2)	0.26559 (6)	0.77544 (11)	0.0435 (3)	
H1A	0.0365	0.2638	0.6951	0.052*	
N2	−0.1342 (2)	0.30720 (6)	0.82213 (11)	0.0422 (3)	
C1	0.4351 (3)	0.18305 (7)	0.68139 (14)	0.0415 (4)	
C2	0.3961 (3)	0.18397 (7)	0.80733 (13)	0.0385 (3)	
C3	0.5525 (3)	0.14165 (7)	0.89334 (15)	0.0493 (4)	
H3	0.5312	0.1417	0.9778	0.059*	
C4	0.7373 (3)	0.09972 (8)	0.85745 (16)	0.0560 (4)	
H4	0.8409	0.0723	0.9171	0.067*	
C5	0.7674 (3)	0.09876 (8)	0.73201 (16)	0.0549 (4)	
H5	0.8892	0.0699	0.7065	0.066*	
C6	0.6191 (3)	0.14000 (8)	0.64527 (15)	0.0508 (4)	
H6	0.6417	0.1392	0.5611	0.061*	
C7	0.2058 (3)	0.22765 (7)	0.85884 (13)	0.0397 (4)	
C8	−0.2876 (3)	0.34231 (7)	0.74000 (15)	0.0483 (4)	
H8	−0.2834	0.3378	0.6539	0.058*	
C9	−0.4676 (3)	0.38898 (7)	0.77975 (14)	0.0460 (4)	
C10	−0.5197 (3)	0.40836 (8)	0.89143 (17)	0.0582 (5)	
H10	−0.4375	0.3928	0.9714	0.070*	
C11	−0.7221 (4)	0.45682 (9)	0.8659 (2)	0.0668 (5)	
H11	−0.7991	0.4793	0.9254	0.080*	
C12	−0.7803 (4)	0.46390 (9)	0.7418 (2)	0.0767 (6)	
H12	−0.9090	0.4928	0.6988	0.092*	

### Atomic displacement parameters (Å^2^)

**Table:**

	*U*^11^	*U*^22^	*U*^33^	*U*^12^	*U*^13^	*U*^23^
O1	0.0660 (8)	0.0871 (9)	0.0369 (6)	0.0281 (6)	0.0263 (5)	0.0161 (6)
O2	0.0647 (7)	0.0696 (8)	0.0325 (6)	0.0026 (6)	0.0206 (5)	0.0006 (5)
O3	0.0984 (10)	0.0848 (9)	0.0550 (8)	0.0339 (8)	0.0000 (7)	−0.0057 (7)
N1	0.0502 (7)	0.0512 (7)	0.0328 (7)	0.0022 (6)	0.0171 (6)	−0.0049 (6)
N2	0.0457 (7)	0.0457 (7)	0.0386 (7)	−0.0034 (6)	0.0167 (6)	−0.0075 (6)
C1	0.0397 (8)	0.0502 (9)	0.0365 (8)	−0.0013 (7)	0.0120 (6)	0.0019 (7)
C2	0.0386 (8)	0.0434 (8)	0.0350 (7)	−0.0083 (6)	0.0107 (6)	−0.0015 (6)
C3	0.0566 (9)	0.0529 (9)	0.0388 (8)	−0.0027 (8)	0.0103 (7)	0.0013 (7)
C4	0.0582 (10)	0.0533 (10)	0.0538 (10)	0.0060 (8)	0.0046 (8)	0.0067 (8)
C5	0.0525 (10)	0.0530 (10)	0.0612 (11)	0.0058 (8)	0.0162 (8)	−0.0053 (8)
C6	0.0512 (9)	0.0611 (10)	0.0439 (9)	0.0048 (8)	0.0185 (7)	−0.0016 (8)
C7	0.0427 (8)	0.0450 (8)	0.0343 (8)	−0.0099 (7)	0.0146 (6)	−0.0019 (7)
C8	0.0584 (10)	0.0526 (9)	0.0362 (8)	−0.0008 (8)	0.0147 (8)	−0.0076 (7)
C9	0.0475 (9)	0.0461 (9)	0.0439 (9)	−0.0030 (7)	0.0080 (7)	−0.0032 (7)
C10	0.0629 (11)	0.0656 (11)	0.0506 (10)	0.0079 (9)	0.0220 (8)	−0.0004 (8)
C11	0.0634 (12)	0.0592 (11)	0.0856 (15)	0.0003 (9)	0.0337 (11)	−0.0125 (10)
C12	0.0694 (13)	0.0596 (12)	0.0966 (17)	0.0202 (10)	0.0055 (12)	−0.0088 (11)

### Geometric parameters (Å, °)

**Table:**

O1—C1	1.3549 (17)	C3—H3	0.9300
O1—H1	0.8200	C4—C5	1.380 (2)
O2—C7	1.2288 (16)	C4—H4	0.9300
O3—C12	1.359 (2)	C5—C6	1.365 (2)
O3—C9	1.3633 (19)	C5—H5	0.9300
N1—C7	1.3503 (18)	C6—H6	0.9300
N1—N2	1.3737 (16)	C8—C9	1.438 (2)
N1—H1A	0.8600	C8—H8	0.9300
N2—C8	1.2720 (19)	C9—C10	1.334 (2)
C1—C6	1.387 (2)	C10—C11	1.410 (2)
C1—C2	1.3991 (19)	C10—H10	0.9300
C2—C3	1.391 (2)	C11—C12	1.310 (3)
C2—C7	1.492 (2)	C11—H11	0.9300
C3—C4	1.374 (2)	C12—H12	0.9300
			
C1—O1—H1	109.5	C5—C6—C1	120.69 (15)
C12—O3—C9	106.28 (15)	C5—C6—H6	119.7
C7—N1—N2	118.33 (11)	C1—C6—H6	119.7
C7—N1—H1A	120.8	O2—C7—N1	120.95 (13)
N2—N1—H1A	120.8	O2—C7—C2	121.25 (13)
C8—N2—N1	115.98 (12)	N1—C7—C2	117.81 (12)
O1—C1—C6	120.38 (13)	N2—C8—C9	120.25 (13)
O1—C1—C2	119.43 (13)	N2—C8—H8	119.9
C6—C1—C2	120.19 (14)	C9—C8—H8	119.9
C3—C2—C1	117.51 (14)	C10—C9—O3	108.97 (14)
C3—C2—C7	116.76 (12)	C10—C9—C8	135.24 (16)
C1—C2—C7	125.71 (13)	O3—C9—C8	115.78 (14)
C4—C3—C2	122.05 (14)	C9—C10—C11	107.40 (17)
C4—C3—H3	119.0	C9—C10—H10	126.3
C2—C3—H3	119.0	C11—C10—H10	126.3
C3—C4—C5	119.29 (15)	C12—C11—C10	106.37 (17)
C3—C4—H4	120.4	C12—C11—H11	126.8
C5—C4—H4	120.4	C10—C11—H11	126.8
C6—C5—C4	120.24 (15)	C11—C12—O3	110.96 (17)
C6—C5—H5	119.9	C11—C12—H12	124.5
C4—C5—H5	119.9	O3—C12—H12	124.5
			
C7—N1—N2—C8	179.41 (13)	C3—C2—C7—O2	4.9 (2)
O1—C1—C2—C3	−178.33 (13)	C1—C2—C7—O2	−173.54 (14)
C6—C1—C2—C3	1.5 (2)	C3—C2—C7—N1	−175.00 (13)
O1—C1—C2—C7	0.1 (2)	C1—C2—C7—N1	6.6 (2)
C6—C1—C2—C7	179.90 (13)	N1—N2—C8—C9	−177.93 (12)
C1—C2—C3—C4	−0.6 (2)	C12—O3—C9—C10	−0.45 (19)
C7—C2—C3—C4	−179.16 (13)	C12—O3—C9—C8	179.81 (14)
C2—C3—C4—C5	−0.8 (2)	N2—C8—C9—C10	1.6 (3)
C3—C4—C5—C6	1.4 (3)	N2—C8—C9—O3	−178.74 (14)
C4—C5—C6—C1	−0.5 (2)	O3—C9—C10—C11	0.28 (19)
O1—C1—C6—C5	178.84 (14)	C8—C9—C10—C11	179.95 (17)
C2—C1—C6—C5	−1.0 (2)	C9—C10—C11—C12	0.0 (2)
N2—N1—C7—O2	1.8 (2)	C10—C11—C12—O3	−0.3 (2)
N2—N1—C7—C2	−178.36 (11)	C9—O3—C12—C11	0.5 (2)

### Hydrogen-bond geometry (Å, °)

**Table:**

*D*—H···*A*	*D*—H	H···*A*	*D*···*A*	*D*—H···*A*
O1—H1···O2^i^	0.82	2.14	2.804 (2)	139
N1—H1A···O1	0.86	1.99	2.650 (2)	133

Symmetry codes: (i) *x*+1/2, −*y*+1/2, *z*−1/2.
